# Molecular Epidemiology and Evolutionary Trajectory of Emerging Echovirus 30, Europe

**DOI:** 10.3201/eid2706.203096

**Published:** 2021-06

**Authors:** Kimberley S.M. Benschop, Eeva K. Broberg, Emma Hodcroft, Dennis Schmitz, Jan Albert, Anda Baicus, Jean-Luc Bailly, Gudrun Baldvinsdottir, Natasa Berginc, Soile Blomqvist, Sindy Böttcher, Mia Brytting, Erika Bujaki, Maria Cabrerizo, Cristina Celma, Ondrej Cinek, Eric C.J. Claas, Jeroen Cremer, Jonathan Dean, Jennifer L. Dembinski, Iryna Demchyshyna, Sabine Diedrich, Susanne Dudman, Jake Dunning, Robert Dyrdak, Mary Emmanouil, Agnes Farkas, Cillian De Gascun, Guillaume Fournier, Irina Georgieva, Ruben Gonzalez-Sanz, Jolanda van Hooydonk-Elving, Anne J. Jääskeläinen, Ruta Jancauskaite, Kathrin Keeren, Thea K. Fischer, Sidsel Krokstad, Lubomira Nikolaeva–Glomb, Ludmila Novakova, Sofie E. Midgley, Audrey Mirand, Richard Molenkamp, Ursula Morley, Joël Mossong, Svajune Muralyte, Jean-Luc Murk, Trung Nguyen, Svein A. Nordbø, Riikka Österback, Suzan Pas, Laura Pellegrinelli, Vassiliki Pogka, Birgit Prochazka, Petra Rainetova, Marc Van Ranst, Lieuwe Roorda, Isabelle Schuffenecker, Rob Schuurman, Asya Stoyanova, Kate Templeton, Jaco J. Verweij, Androniki Voulgari-Kokota, Tytti Vuorinen, Elke Wollants, Katja C. Wolthers, Katherina Zakikhany, Richard Neher, Heli Harvala, Peter Simmonds

**Affiliations:** National Institute for Public Health and the Environment, Bilthoven, the Netherlands (K.S.M. Benschop, D. Schmitz, J. Cremer);; European Centre for Disease Prevention and Control, Stockholm, Sweden (E.K. Broberg);; Biozentrum, University of Basel and Swiss Institute of Bioinformatics, Basel, Switzerland (E. Hodcroft, R. Neher);; Karolinska University Hospital and Karolinska Institute, Stockholm (J. Albert, R. Dyrdak);; Cantacuzino, Bucharest, Romania (A. Baicus);; CHU Clermont-Ferrand, National Reference Centre for Enteroviruses and Parechoviruses, Clermont-Ferrand, France (J. Bailly, A. Mirand);; Landspitali-National University Hospital, Reykjavik, Iceland (G. Baldvinsdottir);; National laboratory of Health, Environment and Food, Ljubljana, Slovenia (N. Berginc);; National Institute for Health and Welfare, Helsinki, Finland (S. Blomqvist);; Robert-Koch-Institue, Berlin, Germany (S. Böttcher S. Diedrich, K. Keeren);; The Public Health Agency of Sweden, Solna, Sweden (M. Brytting, K. Zakikhany);; National Public Health Center, Budapest, Hungary (E. Bujaki, A. Farkas);; Instituto de Salud Carlos III, Madrid, Spain (M. Cabrerizo, R. Gonzalez-Sanz);; Public Health England, Colindale, UK (C. Celma, J. Dunning);; University of Oslo and Oslo University Hospital, Oslo, Norway (S. Dudman);; Charles University, Prague, Czech Republic (O. Cinek);; Leiden University Medical Center, Leiden, the Netherlands (E.C.J. Claas);; University College Dublin, Dublin, Ireland, UK (J. Dean, C. De Gascun, U. Morley);; World Health Organization National Polio Entero Reference Laboratory, Norwegian Institute of Public Health, Oslo (J.L. Dembinski, S. Dudman);; Public Health Center of the Ministry of Health of Ukraine, Kiev, Ukraine (I. Demchyshyna);; Hellenic Pasteur Institute, Athens, Greece (M. Emmanouil, V. Pogka, A. Voulgari-Kokota);; Laboratoire National de Santé, Dudelange, Luxembourg (G. Fournier, T. Nguyen, J. Mossong);; National Center of Infectious and Parasitic Diseases, Sofia, Bulgaria (I. Georgieva, L. Nikolaeva-Glomb, A. Stoyanova);; Microvida, Breda, the Netherlands (J. van Hooydonk-Elving, S. Pas);; University of Helsinki and Helsinki University Hospital, Helsinki (A.J. Jääskeläinen);; National Public Health Surveillance Laboratory, Vilnius, Lithuania (R. Jancauskaite, S. Muralyte);; Nordsjaellands University Hospital, Hilleroed, Denmark (T.K. Fischer);; Statens Serum Institute and University of Copenhagen, Copenhagen, Denmark (T.K. Fischer);; University Hospital of Trondheim, Norway (S. Krokstad, S.A. Nordbø);; National Institute of Public Health, Prague (L. Novakova, P. Rainetova);; Danish WHO National Reference Laboratory for Poliovirus, Statens Serum Institut, Copenhagen (S.E. Midgley);; Erasmus Medical Center, Rotterdam, the Netherlands (R. Molenkamp);; Elisabeth Tweesteden Hospital, Tilburg, the Netherlands (J.-L. Murk, J.J. Verweij);; Norwegian University of Science and Technology, Trondheim (S.A. Nordbø);; Turku University Hospital, Turku, Finland (R. Österback, T. Vuorinen);; University of Milan, Milan, Italy (L. Pellegrinelli);; Austrian Agency for Health and Food Safety, Vienna, Austria (B. Prochazka); Rega Institute KU Leuven, Leuven, Belgium (M. Van Ranst, E. Wollants);; Maasstad Ziekenhuis, Rotterdam (L. Roorda);; Centre de Biologie Est des Hospices Civils de Lyon, Lyon, France (I. Schuffenecker);; University Medical Center Utrecht, Utrecht, the Netherlands (R. Schuurman); N; ational Health Services Scotland, Edinburgh, Scotland, UK (K. Templeton);; University of Turku, Turku (T. Vuorinen) Amsterdam University Medical Center, Amsterdam, the Netherlands (K.C, Wolthers);; University College London, London, UK (H. Harvala);; National Health Service, Colindale (H. Harvala); University of Oxford, Oxford, UK (P. Simmonds)

**Keywords:** Molecular epidemiology, evolutionary trajectory, epidemiological monitoring, whole-genome sequencing, genetic recombination, neurological manifestations, enterovirus, echovirus 30, meningitis/encephalitis, European Non-Polio Enterovirus Network, Nextstrain, viruses, Europe

## Abstract

In 2018, an upsurge in echovirus 30 (E30) infections was reported in Europe. We conducted a large-scale epidemiologic and evolutionary study of 1,329 E30 strains collected in 22 countries in Europe during 2016–2018. Most E30 cases affected persons 0–4 years of age (29%) and 25–34 years of age (27%). Sequences were divided into 6 genetic clades (G1–G6). Most (53%) sequences belonged to G1, followed by G6 (23%), G2 (17%), G4 (4%), G3 (0.3%), and G5 (0.2%). Each clade encompassed unique individual recombinant forms; G1 and G4 displayed >2 unique recombinant forms. Rapid turnover of new clades and recombinant forms occurred over time. Clades G1 and G6 dominated in 2018, suggesting the E30 upsurge was caused by emergence of 2 distinct clades circulating in Europe. Investigation into the mechanisms behind the rapid turnover of E30 is crucial for clarifying the epidemiology and evolution of these enterovirus infections.

Echovirus 30 (E30) is a common cause of viral meningitis outbreaks and upsurges reported worldwide (*1*–*6*). In 2018, E30 circulation was high, and large-scale E30 meningitis-related upsurges were reported in Denmark, Germany, the Netherlands, Norway, and Sweden, compared with data collected during 2015–2017 (*2*). E30 was detected in 14.5% of all confirmed enterovirus cases (*2*). The virus affected mainly children 0–4 years of age and adults 26–45 years of age, and 75% of cases had central nervous system involvement (*2*).

E30 is classified into the *Enterovirus B* (EV-B) species within the *Picornaviridae* family of human enteroviruses and is divided into 2 genogroups (GG), I and II (*7*). Most currently circulating strains are classified as GGII (*7*,*8*). The genome (positive-sense single-stranded RNA) is ≈7.4 kb long and contains 5′ and 3′ untranslated regions (UTRs) flanking a single open reading frame (ORF), encoding 4 structural proteins (viral protein [VP] 0, VP2, VP3, and VP1) and 7 nonstructural proteins (NSP; 2A, 2B, 2C, 3A, 3B [also known as VPg], 3C, and 3D polymerase [3Dpol]).

E30 outbreaks display a cyclic incidence pattern of 3–5 years (*1*,*7*,*9*–*13*). Typically, outbreaks and upsurges are associated with rapid spread of different, relatively short-lived, strains defined by VP1 capsid gene sequences (*1*,*7*,*8*,*14*–*16*). Novel E30 variants have invariably undergone recombination with other EV-B types before their emergence. Recombination results in the generation of novel recombinant forms (RFs) that are chimaeras of E30-derived structural genes with NSP, 5′ UTR sequences, or both, which are derived from cocirculating E30 strains or other EV-B types, such as E9 and E11 (*10*–*12*,*14*,*17*,*18*). The role of VP1 sequence change, recombination, and other factors driving phenotypic changes in virus transmissibility or pathogenicity, and the contributions of changes in population immunity, are crucial for clarifying the underlying causes of E30 outbreaks and upsurges in cases (*15*,*19*–*22*).

We performed an in-depth analysis of the genetic diversity of E30 strains detected during a large-scale upsurge in cases in Europe during 2018. We collated sequences obtained by participating laboratories in 22 countries and analyzed the epidemiologic and evolutionary profiles in this molecular study.

## Methods

### Data Collection

An invitation to participate in this study was sent on November 13, 2018, to co-authors of the E30–2018 study (*2*) through the European Centre for Disease Prevention and Control (ECDC) Epidemic Intelligence Information System Vaccine-Preventable Diseases platform (https://www.ecdc.europa.eu/en/publications-data/epidemic-intelligence-information-system-epis), and to members of the European Non-Polio Enterovirus Network (ENPEN; https://www.escv.eu/enpen). We requested pseudonymized data from 2016–2018 with sample identifier, sampling date, specimen type, and sequence in FASTA be sent to ECDC secure file transfer protocol server by January 7, 2019. We also collected optional data, such as patient age, clinical presentation, whether they were hospitalized, and infection outcome. We excluded submissions without virus sequence data (Appendix Figure 1).

### Sequence Data Collection

We requested that the FASTA sequence data contain the VP1 gene and collected 1,784 records (Appendix Figure 1). Sequences were obtained from enterovirus-positive samples by using 5′ UTR PCR (*23*) and typed within the VP1 gene by using Sanger sequencing (*2*,*24*). We excluded sequences with indicators of poor sequence quality, such as >2 ambiguous or undefined bases, in-frame stop codons, identical to reference E30 strains; sequences of the wrong type, such as E3; or sequences shorter than 200 basepairs or spanning a non-VP1 region. In total, we had 1,407 study sequences that comprised 2 nonoverlapping regions, 1,262 sequences from region 1 (nt positions 2543–2902, according to the prototype E30 strain Bastianni, GenBank accession no. AF311938) and 145 sequences from region 2 (nt positions 2916–3428). Of these, 1,329 sequences were collected during 2016–2018 and 78 during 2010–2015. We used the 2010–2015 sequences for phylogenetic reconstruction but excluded these from further data analysis (Appendix Figure 1). 

For additional analysis of the 3D polymerase (3Dpol) region, we randomly selected records from each clade to ensure fair distribution of sequence data. We asked participants to send either extracted RNA in a QIAGEN (https://www.qiagen.com) spin column at room temperature for next-generation sequencing (NGS) or to conduct 3Dpol sequencing of the 549 nucleotides, as previously described (*17*).

### Epidemiologic and Statistical Analyses

We descriptively analyzed clinical symptoms and age. Patients were stratified into the following age groups: <3 months, 3–23 months, 2–5 years, 6–15 years, 16–25 years, 26–45 years, 46–65 years, and >65 years. Crude odds ratios with 95% CI were used to express magnitude of association between continuous or categorical variables in multivariate logistic regression.

### Next-Generation Sequencing

Stool suspensions and CSF samples were processed to remove as much nonviral material as possible by using centrifugation, filtration, and endonuclease treatment. RNA was extracted by using the MagNAPure 96 (Roche Diagnostics, https://www.roche.com) automated extraction kit or QIAGEN filters and eluted in 50 µL of elution buffer (Appendix).

Complementary DNA (cDNA) and double stranded DNA (dsDNA) were generated and purified (Appendix). For tagmentation and library preparation, the Nextera XT DNA Library Preparation Kit (Ilumina, https://www.illumina.com) was used according to the manufacturer’s instructions. Runs were performed on the Nextseq (Ilumina). Raw data were processed by using Jovian (D. Schmitz et al., unpub. data, https://github.com/DennisSchmitz/Jovian) (Appendix). 

### Nucleotide Sequences and Phylogenetic Analysis

We conducted VP1 phylogenetic reconstruction with the 1,407 study sequences and 324 sequences extracted from Genbank. We selected region 1 for clade analysis because it is more commonly used for enterovirus typing (*24*). We performed analysis of region 2 sequences based on sequence clustering, in which both region 1 and 2 were available, such as full-length sequences or sequences spanning the entire VP1 gene. Sequencing of the 540 nt 3Dpol gene, positions 5825–6364, also was provided for 12 samples with region 1 sequences (Appendix Figure 1). Sanger sequencing indicated that samples did not display double infection and that VP1 and 3Dpol were from 1 virus. Complete genomes (≈7.3 kb) were generated for 48 sequences by using NGS. To compare 3Dpol groupings within EV species B, we downloaded all sequences available from GenBank as of October 18, 2019, that were >70% complete between positions 5825–6364 with <6 ambiguous base positions and <6 undetermined bases and without stop codons. We aligned the downloaded sequences with complete genomes or 3Dpol sequences from our study.

We aligned data by using sequence editor SSE version 1.3 (http://www.virus-evolution.org). We generated maximum-likelihood and neighbor-joining trees for VP1 and 3Dpol regions by using MEGA version 7 (https://www.megasoftware.net) with the optimal model (general time reversible plus invariant sites plus gamma distribution for rates over sites) and 100 bootstraps (*25*). We analyzed the species B dataset with neighbor-joining and maximum composite likelihood distances.

### Nextstrain VP1 Phylodynamic Analysis

The dataset used for Nextstrain phylodynamic analysis comprised 1,285 sequences; 1,215 study sequences (region 1) and 70 complete VP1 sequences extracted from GenBank (Appendix Figure 1). We excluded sequences shorter than 250 bp, sequences from samples collected before 1958, and sequences deemed as outliers during phylogenetic reconstruction. We deemed these outliers recombinants with possible recombination breakpoints within the sequence fragment used made phylogenetic reconstruction impossible.

We aligned sequences by using MAFFT (*26*). We inferred a phylogenetic tree by using IQ-TREE (*27*) and generated time-resolved trees by using TreeTime (*28*) by estimating the mutation rate. When available, we attached to sequences data on country, sample type, E30 clade, age groups, and clinical data, such as whether patients were hospitalized and their symptoms. We provided the resulting Nextstrain build for viewing (https://nextstrain.org/community/enterovirus-phylo/echo30-2019/vp1).

### Nextstrain VP1:3Dpol Tanglegram Phylodynamics

We used a dataset of 110 sequences to conduct 3Dpol analysis, including 48 complete genome sequences and 12 3Dpol sequences generated in this study and 50 sequences extracted from GenBank (Appendix Figure 1). We aligned 3Dpol sequences to the E30 reference sequence (GenBank accession no. MK238483) and inferred phylogenetic and time-resolved trees as we did for VP1, but we used a fixed clock rate of 4 × 10^−3^ substitutions/site/year during the time-resolved tree reconstruction. We provided the resulting Nextstrain tanglegram build for viewing (https://nextstrain.org/community/enterovirus-phylo/echo30-2019/3D:community/enterovirus-phylo/echo30-2019/vp1) and the codes for both VP1 and 3Dpol analyses (https://github.com/enterovirus-phylo/echo30-2019).

### Genbank and ENA Accession Numbers

We deposited VP1 and complete genome sequences in GenBank under accession nos. KC309427–37, KY986976–7033, MK251835–6, MK372854–80, MK507733–7, MK814991–6288, and MK895104–9 and 3Dpol sequences under accession nos. MN395293–303. We deposited NGS fastq reads in European Nucleotide Archive database under accession nos. SAM17101211–58.

## Results

### Molecular Epidemiology and Demographics

**Figure 1:**
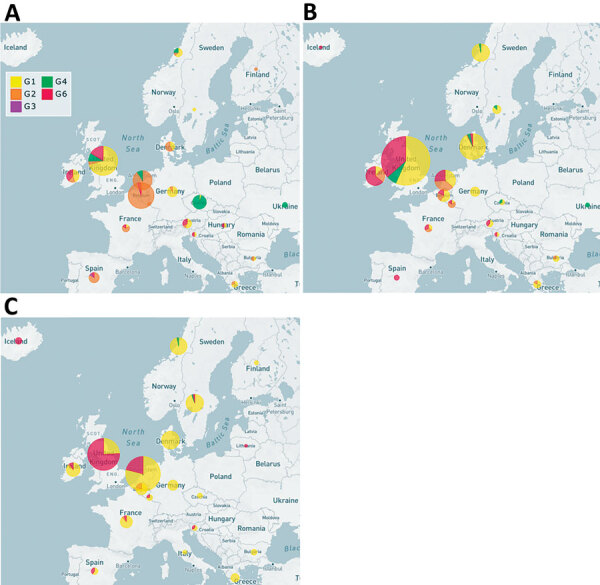
Geographic distribution of echovirus 30 (EV30) clades, Europe, 2016–2018. Clades G1–G6 were detected among 1,329 EV30 cases from 22 countries. A) 2016; B) 2017; C) 2018.

**Figure 2:**
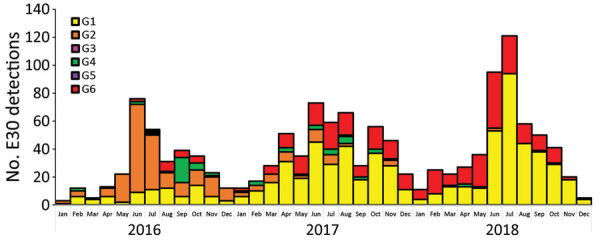
Monthly distribution of echovirus 30 (EV30) clades G1–G6 detected among 1,329 sequences submitted from 22 countries in Europe during 2016–2018.

During 2016–2018, a total of 1,329 E30 records representing 1,292 cases that fulfilled the study criteria were submitted from 22 countries (Table 1; Appendix Figure 1). During those 3 years, the total number of E30 cases steadily increased (Table 1). The numbers varied per country per year, and we noted a clear upsurge in 2018 in several, but not all, countries (Table 1; Figure 1). Of the 1,329 records analyzed, 443 (33%) were from the United Kingdom; the Netherlands submitted 198 (15%) and Spain 162 (12%) records. Other countries submitted from 1 (<1%) to 117 (9%) records. Specimen type was reported for 1,312 (98.7%) records. Most (70%; 924/1,312) samples were cerebrospinal fluid specimens, but other specimen types included 269 (21%) from feces specimens, 102 (8%) from respiratory, and 17 (1%) from blood. During the study period, E30 records were submitted more frequently in summer months; 18.4% (n = 244) were submitted in June, 17.6% in July (n = 234), and 11.7% in August (n = 155) (Figure 2).

**Table 1:** Number of echovirus 30 records with curated viral protein 1 sequences by country, 2016–2018*

Country	2016, n = 325	2017, n = 493	2018, n = 511	Total, n = 1,329
Austria	6 (1.8)	3 (0.6)	0	9 (0.7)
Belgium	74 (22.8)	2 (0.4)	15 (2.9)	91 (6.8)
Bulgaria	0	4 (0.8)	4 (0.8)	8 (0.6)
Czech Republic	21 (6.5)	2 (0.4)	3 (0.6)	26 (2.0)
Germany	11 (3.4)	4 (0.8)	12 (2.3)	27 (2.0)
Denmark	7 (2.2)	73 (14.8)	37 (7.2)	117 (8.8)
Spain	86 (26.5)	37 (7.5)	39 (7.6)	162 (12.2)
Finland	1 (0.3)	0	2 (0.4)	3 (0.2)
France	4 (1.2)	2 (0.4)	17 (3.3)	23 (1.7)
Greece	0	3 (0.6)	8 (1.6)	11 (0.8)
Hungary	2 (0.6)	0	0	2 (0.2)
Ireland	13 (4.0)	46 (9.3)	23 (4.5)	82 (6.2)
Iceland	0	0	5 (1.0)	5 (0.4)
Italy	0	0	2 (0.4)	2 (0.2)
Lithuania	0	0	1 (0.2)	1 (0.1)
Luxembourg	0	4 (0.8)	4 (0.8)	8 (0.6)
Netherlands	33 (10.2)	23 (4.7)	142 (27.8)	198 (14.9)
Norway	4 (1.2)	28 (5.7)	34 (6.7)	66 (5.0)
Sweden	0	0	36 (7.0)	36 (2.7)
Slovenia	1 (0.3)	0	3 (0.6)	4 (0.3)
Ukraine	3 (0.9)	2 (0.4)	0	5 (0.4)
United Kingdom	59 (18.2)	260 (52.7)	124 (24.3)	443 (33.3)

*All values expressed as no. (%).

Age was available for 1,080 (83.6%) cases and ranged from 0 to 73 years with a mean age of 18.7 years. Children <5 years of age (n = 360, 33.3%) and adults 26–45 years of age (n = 409, 37.9%) were most affected (Table 2). Infants <3 months of age also were heavily affected (n = 223 cases, 20.6%) (Table 2).

**Table 2:** Distribution of echovirus 30 cases in Europe by age group and clade*

Clade	Age range	Total no. (%)	Mean age, y (95% CI)	p value

<3 mo	3–23 mo	2–5 y	6–15 y	16–25 y	26–45 y	46–65 y	>65 y
G1	124 (55.6)	24 (54.5)	30 (33.3)	58 (43.6)	84 (56.8)	227 (55.9)	18 (0.6)	3 (37.5)	568 (52.6)	19.24 (17.94–20.54)	Referent
G2	41 (18.4)	10 (22.7)	29 (31.2)	23 (17.3)	7 (4.7)	32 (7.9)	2 (6.7)	1 (12.5)	145 (13.4)	12.07 (9.55–14.58)	<0.001
G3	0	0	0	0	0	2 (0.4)	0	0	2 (0.2)	35.5 (29.15–41.85)	0.142
G4	9 (4.0)	0	4 (4.3)	17 (12.8)	11 (7.4)	13 (3.2)	2 (6.7)	0	56 (5.2)	16.82 (13.06–20.57)	0.269
G5	0	0	0	0	0	1 (0.2)	0	0	1 (0.1)	36.84 (NA)	0.260
G6	49 (22.0)	10 (22.7)	30 (32.3)	35 (26.3)	43 (29.0)	134 (33.0)	8 (26.7)	4 (50.0)	308 (28.5)	21.11 (19.34–22.88)	0.090
Total	223	44	93	133	145	449	30	8	1,080	18.73 (17.78–19.68)	0.001

*Values are no. (%) except where otherwise indicated. NA, not applicable.

Clinical information was available for 734 (56.8%) E30 cases, of which 380 cases had unknown symptomology. For most (28.7%, n = 211) cases, the recorded signs and symptoms suggested meningitis. Symptoms of acute flaccid paralysis were reported in 1 case, encephalitis in 3 cases, and meningoencephalitis in 8 cases. Fever, either as sole symptom or in combination with other signs and symptoms, was recorded in only 52 (7.1%) cases. Unfortunately, not all records were filled in completely, and clinical data were absent for some samples. Other signs and symptoms mentioned were gastrointestinal symptoms in 6 cases, respiratory symptoms in 6, rash in 2, other neurologic symptoms in 4, or other unspecified in 53 cases; 8 cases had no symptoms. We created an interactive representation of age and clinical features of sequences from E30 cases, which we made available on Nextstrain (https://nextstrain.org/community/enterovirus-phylo/echo30-2019/vp1).

Hospitalization status was available for only 17.6% (n = 228) of cases, only 5 of which had no hospitalization. The low fraction of hospitalization reported limited further analysis. No deaths were reported.

### E30 Phylodynamics 

**Figure 3:**
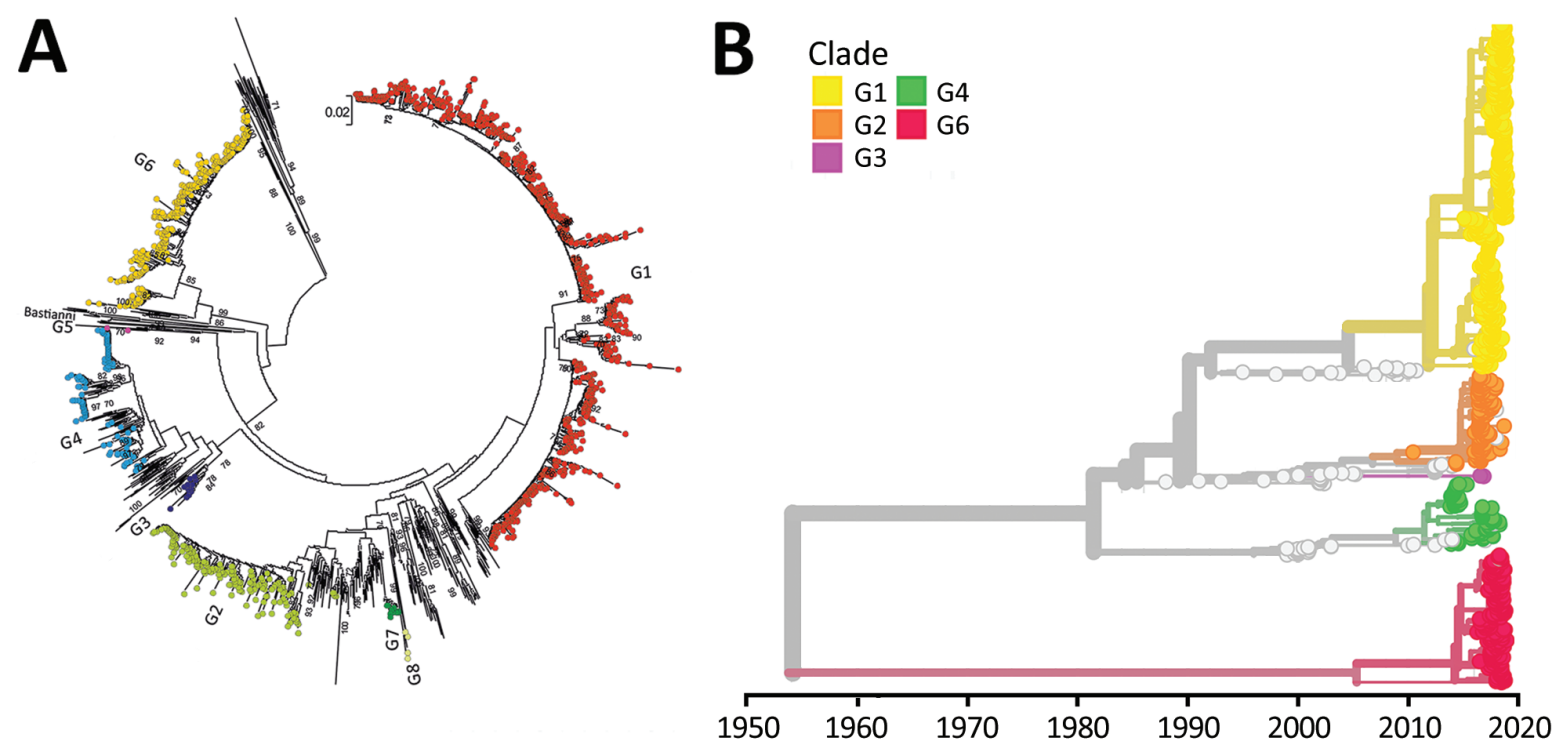
Phylodynamic analysis of region 1 in a curated study of echovirus 30 (E30) viral protein 1 (VP1) sequences from 22 countries in Europe, 2010–2018. We constructed the bootstrapped maximum likelihood neighbor-joining trees using 47 full length sequences and 277 VP1 sequences extracted from GenBank. E30 groups 1–8 are labeled. A) Maximum likelihood trees constructed by using MEGA version 7.0 (https://www.megasoftware.net). Prototype E30 strain Bastianni, (GenBank accession no. AF311938) was used as a reference. Scale bar indicates nucleotide substitutions per site. B) Maximum likelihood trees constructed by using Nextstrain (https://nextstrain.org) from which we dropped several problematic sequences, including group 5.

Among the 1,329 curated VP1 study sequences, 1,019 (76.7%) could be subdivided into 5 distinct clades, G1–G5, that showed >5% sequence divergence from one another (Figure 3, panel A). The mean divergence between VP1 nucleotide sequences of G1–G5 was 12.4%–15.2%, which translated to 2.8%–3.9% amino acid sequence divergence. Most (704, 53%) sequences belonged to G1, but 229 (17.2%) were in G2, 59 (4.4%) in G4, 4 (0.3%) in G3, and 2 (0.2%) in G5. These sequences all were assigned to GGII, 1 of 2 previously reported genogroups (*7*). The remaining 310 VP1 sequences formed a single clade, G6 (Figure 3), showing 20.6% mean nucleotide differences and 8.5% amino acid differences from the VP1 sequences within G1–G5 clades. G6 was sufficiently divergent from G1–G5 (GGII). The divergence falls within the nucleotide divergence between GGI–GGII (19%–22%) (*7*), and G6 can be considered a third genogroup, GGIII.

The phylogeny showed a rapid turnover of E30 clades over the 3 years sampled, shifting from G2 dominating in 2016 to G1 and G6 dominating in 2017 and 2018 (Figure 1). In 2016, 58.5% of strains were G2, and this genotype was identified in 11/22 (50%) countries. G2 was detected in only 8 countries in 2017 and only 4 countries in 2018. Similarly, G4 disappeared during 2016–2018. In 2016, both G1 and G6 were detected, G1 in 24.6% (n = 80) of virus strains in 10 countries and G6 in 6.5% (n = 21) of virus strains in 7 countries. Rates of detection for G1 and G6 steadily increased in 2017; G1 was detected in 59.2% (n = 292) of virus strains in 12 countries and G6 in 26.4% (n = 130) of virus strains in 7 countries (Figure 1). During the 2018 upsurge, 64.6% (330) of sequences reported in 17 countries belonged to G1, and 33.9% (173) in 11 countries belonged to G6 (Figure 1). These data indicate the occurrence of >2 distinct viruses dominating the upsurge in 2018 (Figure 1).

We used Nextstrain to create an interactive phylodynamic tree and map to explore relationships of the E30 study VP1 sequences in G1–G6 (https://nextstrain.org/community/enterovirus-phylo/echo30-2019/vp1) (Figure 3, panel B; Appendix Figure 2). We deemed G5 sequences as outliers and did not include these during phylogenetic reconstruction. Molecular clock analysis of the VP1 region revealed an estimated substitution rate of 5.12 × 10^−3^ substitutions/per site/per year, comparable to rates previously determined for a range of enteroviruses.

Most E30 G1 viruses were detected among infants <3 months of age (135/568, 24%) and in young adults 26–45 years of age (227/568, 40%) (Table 2). G2 (100/145, 68%) and G4 (29/59, 52%) were most frequent among children 3 months–15 years of age. G6 mainly was detected among children 3 months–15 years of age (100/308, 32.5%) and in adults 26–45 years of age (134/308, 43.5%). Only 2 cases of G3 and 1 of G5 were reported with age information (Table 2).

### Amino Acid Diversity

Most E30 VP1 sequences within clades G1, G2, G4, and G6 displayed specific amino acid substitutions. G6 sequences predominantly displayed amino acid changes at position 56 (Y-F), position 84 within the BC loop (V-A), position 87 within the BC loop (E-D), and position 145 (V/I) compared with G1, G2, and G4. Most G1 and G6 sequences had a valine at positions 54 and 120 compared with the G2 and G4 sequences, which had an isoleucine. At position 122, most G4 sequences contained a leucine, whereas G1, G2, and G6 sequences contained a phenylalanine. Interactive data are available on Nextstrain (https://nextstrain.org/community/enterovirus-phylo/echo30-2019/vp1).

### Recombination Analysis

**Figure 4:**
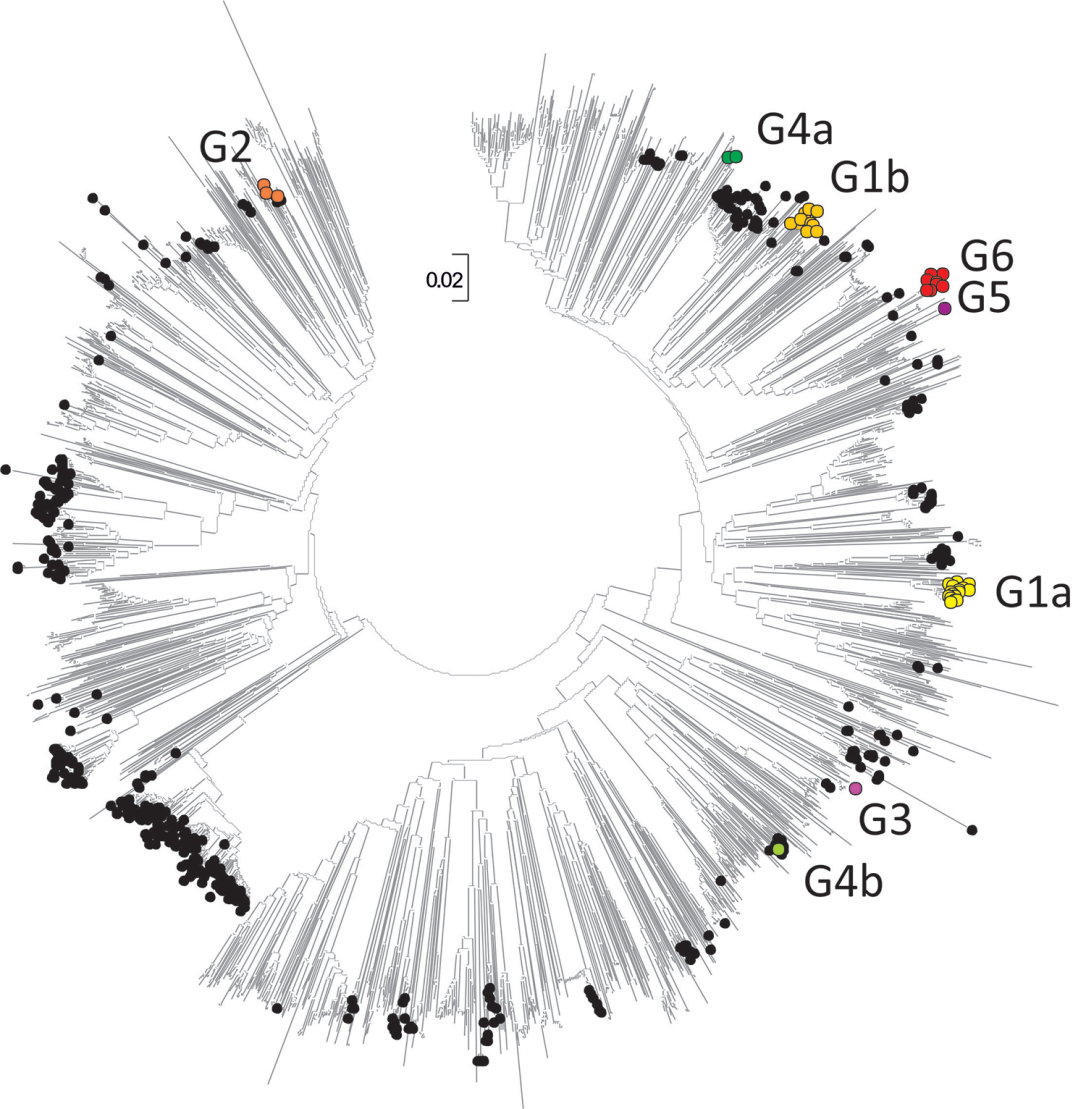
Neighbor-joining tree of 3D polymerase (3Dpol) sequences of echovirus 30 (E30) study samples and sequences from previously described E30 strains. The tree was constructed from Jukes-Cantor corrected nucleotide sequence distances in MEGA version 7.0 (https://www.megasoftware.net). Colored circles represent clades G1–G6 from this study; black circles represent 581 previously described E30 strains; and unlabeled branches represent all other species B types (n = 1,566) available in GenBank as of October 18, 2019. Scale bar indicates nucleotide substitutions per site.

**Figure 5:**
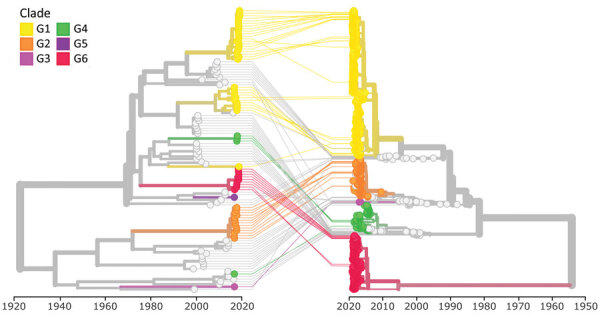
Tanglegram of echovirus 30 (E30) phylogenetic virus protein 1 (VP1) (right) and 3D polymerase (3Dpol) (left) by year of sample collection. We used 110 sequences and rendered the tanglegram by using Nextstrain (https://www.nextstrain.org). Clades G1–G6 are labeled.

We used 110 sequences containing both VP1 and 3Dpol region and complete genome sequences to analyze recombination events between VP1 and the 3′ distal end of the E30 genome (Appendix Figure 1). The E30 3Dpol sequences formed a series of separate clusters interspersed with those of other species B types, indicative of many within-species recombination events during their diversification (Figure 4). We took the entire published sequence dataset and used a nucleotide sequence distance threshold of 8%, based on pairwise sequence comparisons, which divided sequences into distinct groups (Appendix Figure 3), comparable to those derived from a previous analysis of E30 RFs (*17*). Accordingly, species B could be divided into ≈442 RFs, an indication of the frequency and complexity of recombination events occurring during the evolution of this species. We used Nextstrain to generate an interactive tanglegram of VP1 and 3Dpol RFs (https://nextstrain.org/community/enterovirus-phylo/echo30-2019/3D:community/enterovirus-phylo/echo30-2019/vp1) (Figure 5).

We found that 3Dpol sequences of G1–G6 formed 8 recombination groups, which were separated by other published E30 variants and by other species B types (Figure 5). We noted that viruses within most VP1 clades were monophyletic in 3Dpol, but that G1 and G4 each had undergone further recombination (Figure 5; Appendix Figure 4), a split corresponding to the sublineages evident in the VP1-based tree. The split was identified as a time-related phenomenon, with G1 circulating in 2018 representing a different RF from G1 circulating during 2016 and 2017.

## Discussion

A large upsurge of E30 infections was reported in several countries in Europe during 2018 (*2*). We conducted a comprehensive molecular characterization of E30 by using VP1, 3Dpol, and whole genome sequences. Our molecular characterization enabled an analysis of the recombination events occurring during E30 diversification in Europe, which can be conducted only when dealing with a single infection. Our study used a large EV sequence dataset collected worldwide, comprising 1,329 E30 sequences collected from 22 countries in Europe during 2016–2018 and was made possible due to the large-scale collaboration between countries through ENPEN and ECDC.

The data clearly demonstrate that analysis based on phylogenetic clade assignment shows differential dominance of many different clades. The upsurge in 2018 was caused by appearance of several different clades or genogroups of E30 viruses; G1 in GGII (*7*) and G6 in a novel genogroup, GGIII, which we propose in this study. Viruses from both clades had been circulating for >2 years. In total, 6 clades were identified during the study period and circulated in a pattern of rapid turnover of newly emerging genetic lineages and RFs and their relatively rapid disappearance over time, a pattern that is typical for other enteroviruses (*1*,*7*,*10*,*12*,*13*,*16*–*18*,*29*). In this study, G2 predominated in 2016 and 2017 in central Europe and were subsequently replaced by the G1 and G6 in 2018 (Figures 1, 2). This genetic turnover and the associated string of recombination events during lineage diversification occurred within the 2- to 5-year cyclical pattern of E30 incidence. As expected, each VP1 group corresponded to a separate RF, but G1 underwent a further recombination event as the virus diversified from a common ancestor dated to around 2011 and G4 underwent a further recombination from an ancestor around 2008. Of note, clade G1 showed a time related split in which G1 sequences circulating in 2018 emerged from those circulating in 2016–2017, coinciding with a recombination generating a novel RF. The absence of G3 and G5 sequences in the study population might reflect a generally lower circulation of these strains or perhaps a period of relative quiescence during the survey period. Long-term surveillance is essential to monitor for potential emergence of these strains in future incidence cycles.

The cocirculation of different E30 clades during the 2018 upsurge and in previous years argues against the idea that the periodic emergence of E30 occurs through the evolution of more pathogenic or transmissible forms of the virus. The cocirculation of several different groups fits better with changes in population susceptibility from birth cohort effects and a breach of a critical immunity level that controls E30 spread within the population (*19*). The high susceptibility is reflected by the high number of infected infants, who would have no immunity, and adults whom we hypothesize have no or waning immunity. However, another possibility is that the appearance of several, potentially convergent, amino acid substitutions in VP1 among different E30 groups represented a form of antigenic selection for escape from existing population immunity. The clustering of sites under selection in the BC loop associated with receptor interactions is consistent with this possibility. Serologic studies are required to explore this hypothesis.

As shown in the original description of the upsurge (*2*), a high percentage of cases showed central nervous system involvement, particularly for infants 0–3 months of age and adults 25–44 years of age, consistent with previous observations (*2*,*30*–*32*). The distribution of E30 clades varied among age groups; most infections in infants <3 months of age were caused by G1 and symptomatology varied from fever to acute flaccid paralysis. However, analysis of the clinical correlates was limited by incomplete reporting; only 30% of reported E30 infections included history of symptoms, which hampered comparisons of clinical presentation between different clades. Another limitation is the retrospective study design and bias toward severe and hospitalized cases.

Using Nextstrain, we visualized the various categories of demographic and clinical data, clades, and RFs. Unfortunately, G5 could not be inferred due to possible recombination events within the fragment. Complete reconstruction of E30 temporal events with geographic spread was hampered by the inevitably uneven sampling and testing in different years by the different contributing countries.

This study underpins the strength of the ENPEN consortium, which brings together virologists, public health experts, infectious disease doctors, and scientists across Europe to enable rapid detection and early warning through standardized surveillance. Previous studies using Nextstrain with 2 EV-D68 datasets have shown the value of combining demographic and phylogenetic analysis, both as retrospective (*33*) and real-time analysis (*34*). The E30 dataset and the 2 EV-D68 datasets (*33*,*34*) available on Nextstrain represent large nonpolio enterovirus datasets that support real-time tracking of viruses over time and across countries. These data are of considerable value in infection containment and control of nonpolio enteroviruses.

Differences in surveillance systems, case definitions, and sample selection between institutes and countries make standardized data collection difficult, particularly for denominator data. The differences in data collection proved to be a limitation in our study, and the extent of the circulation of the different strains remains unknown. The emergence and disappearance of viruses from different clades across the years suggests that some form of predictive modeling might be undertaken if data were standardized and provided in real-time through networks such as ENPEN. 

The mechanisms underlying the complex cyclic pattern of E30 and other enteroviruses and the effects of changing population immunity, antigenic changes, virus diversification, pathogenicity, and recombination need further exploration. The emergence of different enterovirus types, and their associated periodicities and population penetrance, might be driven by multiple mechanisms (*19*), making outbreak and upsurge prediction complex. However, continued structured surveillance can clarify enterovirus circulation and evolution and slowly aid in unraveling the complex nature of enteroviruses. 

Appendix Additional information on molecular epidemiology and evolutionary trajectory of recent echovirus 30 emergence, Europe.
