# Supplementary material for: Molecular Epidemiology and Evolutionary Trajectory of Emerging Echovirus 30, Europe
Source: Emerg Infect Dis. 2021 Jun;27(6):1616–26. doi: 10.3201/eid2706.203096 (PMC8153861; doi:10.3201/eid2706.203096)
Supplement: Appendix — Additional information on molecular epidemiology and evolutionary trajectory of recent echovirus 30 emergence, Europe. [file 20-3096-Techapp-s1.pdf]

Article DOI: https://doi.org/10.3201/eid2706.203096

Molecular Epidemiology and Evolutionary Trajectory of Emerging Echovirus 30, Europe

Appendix

Supplementary Methods

Next Generation Sequencing

For stool samples, 5%–15% stool suspension was prepared in 1 mL cell culture medium (MEM with gentamicin) and centrifuged for 5 min at 16,000 RCF. Stool suspensions and cerebrospinal fluid (CSF) samples were centrifuged through Costar Spin-X (Corning, https://www.sigmaaldrich.com) centrifuge tube filters with 0.45 µmol cellulose acetate membrane for 6 min at 32,000 RCF at 4°C. We added 200 µL filtrate to 1.25 µL OmniCleave Endonuclease (Lucigen, https://www.lucigen.com) and 25 µL 25mmol magnesium chloride and incubated at 37°C for 1 h. RNA was extracted by automated extraction using MagNA Pure 96 DNA and Viral NA small volume kits (Roche Diagnostics, https://diagnostics.roche.com) and eluted in 50 µL elution buffer. RNA sent through QIAGEN (https://www.qiagen.com) filters were eluted in 50 µL elution buffer.

For mixture A, cDNA was generated by preparing a 5 µL mixture containing 20 mmol each of dNTP (Roche, https://www.roche.com) and 100 µmol Random Primer 9 (New England Biolabs, https://www.neb.com) to which 11 µL of extracted RNA was added. The mixture was incubated at 85°C for 2 min, and placed on ice for 2 min. Mixture A was added to a second mixture (mixture B), 9 µL containing 1× SuperScript III Reaction Buffer (Invitrogen, https://www.thermofisher.com), 0.125 mol dithiothreitol (DTT; Invitrogen), 50 U RNasin (Invitrogen), and 250 U SuperScript III Reverse Transcriptase (Invitrogen). The final mixture was incubated at 20°C for 5 min, then at 50°C for 50 min, and at 85°C for 5 min. Double stranded (ds)DNA was synthesized in a 100 µL reaction mix (mixture C) containing 20 µL cDNA, 1× NEBNext Second Strand Reaction (New England Biolabs) and 15 µL NEBnext Second Strand Synthesis Enzym Mix (New England Biolabs). The mixture was incubated for 60 min at 16°C, then for 2 min at 30°C, and then for 10 min at 70°C.

dsDNA was purified and concentrated by using the DNA Clean & Concentrator-5 kit (Zymo Research, https://www.zymoresearch.com) according to manufacturer's instructions and eluted in 12 µL. For tagmentation and library preparation, the Nextera XT DNA Library Preparation Kit (Ilumina, https://www.illumina.com) was used according to manufacturer's instructions. Concentrations were measured by using the Kapa Library Quantification Kit (Kapa Biosystems, Inc., https://www.sigmaaldrich.com). Libraries were normalized to 4 nmol with 1.5% PhiX. Samples were tested individually and spread across 4 runs performed on the Nextseq (Illumina), with the NextSeq 500/550 Mid Output Kit version 2.5 (300 cycles) kit (Ilumina). A negative control is included in every run and analyzed via Jovian (https://github.com/DennisSchmitz/Jovian) using default settings (see details below), but no echovirus 30 (E30) hits were found. Sample contamination from 1 run to another run was safeguarded by switching barcodes every 4 runs so that no consecutive run used the same barcode.

The raw data were processed by using Jovian. In brief, Jovian removes human data and low-quality reads. Reads were assembled via metaSPAdes (Center for Algorithmic Biotechnology, cab.spbu.ru/software/meta-spades) and individual contigs are assembled into larger scaffolds, often complete genomes, which are cross referenced via megaBLAST in the NCBI blast NT database (ftp://ftp.ncbi.nlm.nih.gov/blast/db). This database is updated to the latest version every weekend. Taxonomic classification of scaffolds is determined up to species level with a lowest-common ancestor analysis via MGkit (https://mgkit.readthedocs.io/en/0.3.4/introduction.html). Scaffolds of the *Picornaviridae* family are automatically submitted to the enterovirus typing tool and genotyped (https://www.rivm.nl/mpf/typingtool/enterovirus). When no full E30 genome was assembled, scaffolds were manually assembled and curated in SSE version 1.3 (http://www.virus-evolution.org/Downloads/Software).

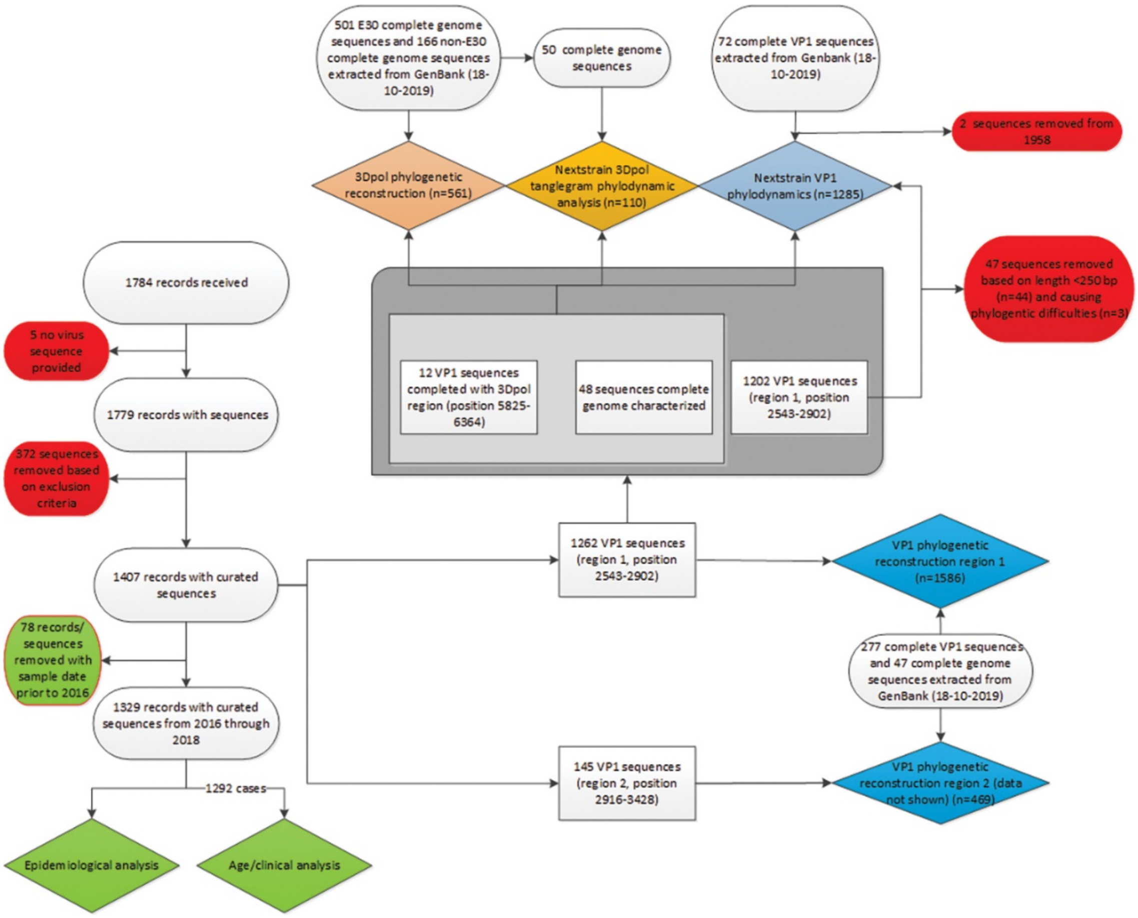

Appendix Figure 1. Diagram of records and sequences used for epidemiologic analysis, phylogenetic reconstruction, and phylodynamic analysis of recent echovirus 30 (E30) emergence, Europe. Green indicates sequences used for epidemiologic analysis. Blue indicates viral protein 1 (VP1) and orange indicates 3D polymerase (3Dpol) used for phylogenetic reconstruction. Light blue indicates VP1 and pink indicates 3Dpol used Nextstrain phylodynamics and tanglegram Red indicates excluded records and sequences.

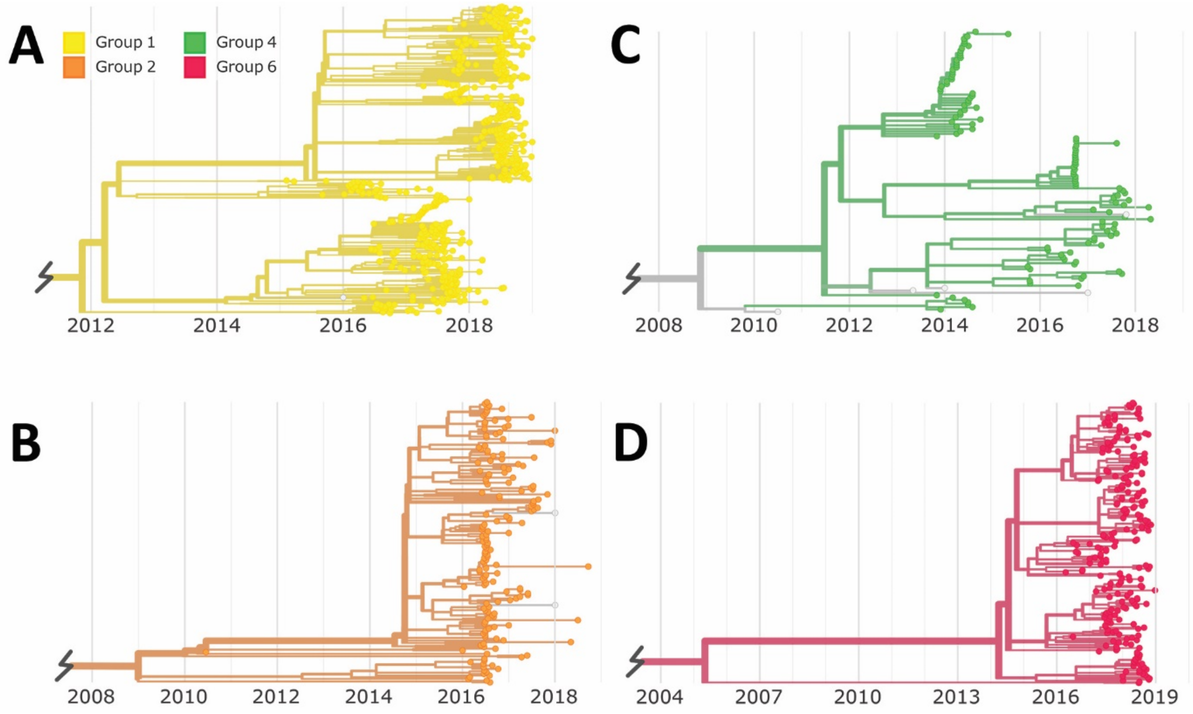

Appendix Figure 2. Phylodynamic analysis of region 1 curated study of viral protein 1 (VP1) echovirus 30 sequences collected during 2010–2018. Major clades are labeled, including group 1 (A); group 2 (B); group 4 (C); and group 6 (D). The 48 full length study sequences and 277 VP1 sequences were extracted from sequences available on GenBank (https://www.ncbi.nlm.nih.gov/genbank) as of October 18, 2019 and constructed and zoomed by using Nextstrain (https://nextstrain.org).

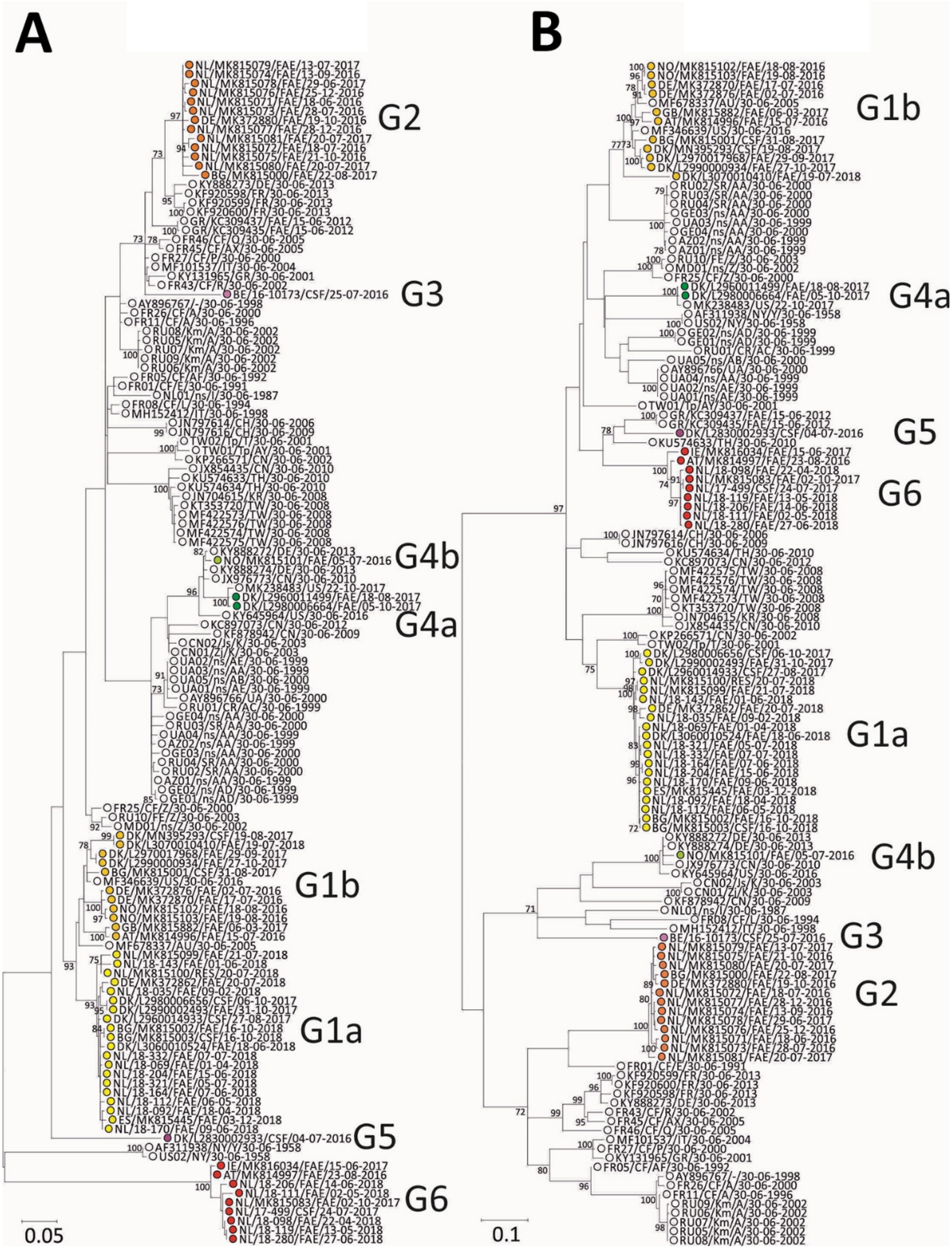

Appendix Figure 3. Maximum likelihood phylogenetic analysis of echovirus 30 (E30) sequences from Europe and globally available sequences. A) Phylogenetic tree of 60 viral protein 1 (VP1) sequences from this study and global sequences. B) Phylogenetic tree of 12 partial 3D polymerase (3Dpol) sequences from this study and global sequences. E30 genetic clades, groups G1–G6, are represented by colored circles; yellow represents G1; orange G2; pink G3; green G4; purple G5; and red G6; white circles represent global strains published in GenBank (https://www.ncbi.nlm.nih.gov/genbank) as of October 18, 2019. The tree was created by using GTR + invariant sites + gamma distribution. Clades with >70% bootstrap support are labeled on the branches. Scale bars indicate nucleotide substitutions per site.

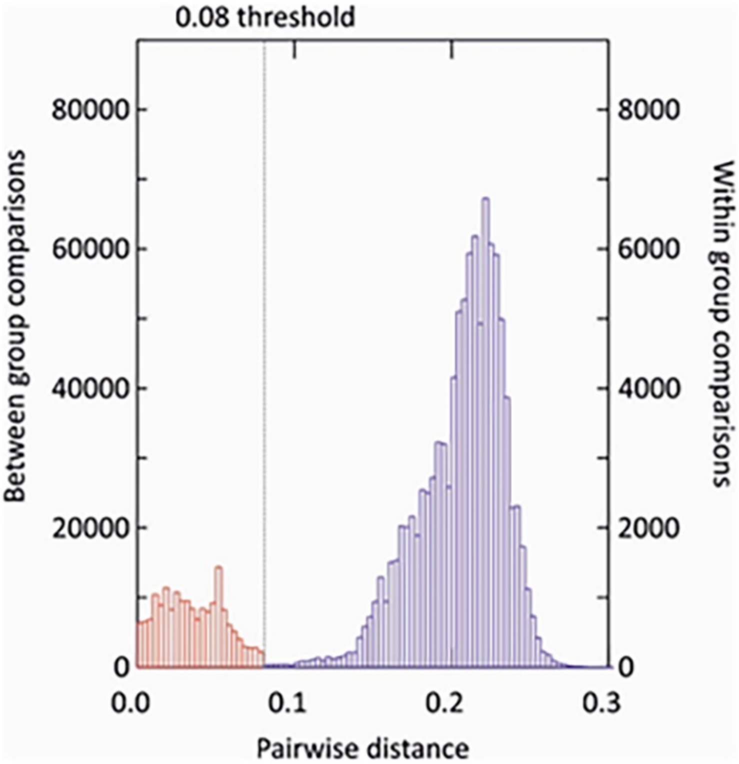

Appendix Figure 4. Pairwise distance distribution within and between 3D polymerase (3Dpol) sequences of echovirus 30, Europe. Red bars demonstrate pairwise distances within members of the same recombinant forms (RFs); blue bars demonstrate differences between members of different RFs. RF assignments are based upon a pairwise nucleotide distance threshold of 0.08.
